# Cloacal feather trimming improves reproductive performance of layer breeder roosters^☆^

**DOI:** 10.1016/j.psj.2025.105737

**Published:** 2025-08-25

**Authors:** Eder O. Barbosa, Regina C.A. Porto, Marcel H. Blank, Felipe L.K. Neto, Ricardo J.G. Pereira

**Affiliations:** a Mercoaves Comercio de Aves Ltda, Tietê, SP, Brazil; b Study Group for Avian Multiplication – GEMA, Department of Animal Reproduction, College of Veterinary Medicine and Animal Sciences, University of São Paulo, Pirassununga, Brazil

**Keywords:** Poultry, Chicken, Management, Male, Fertility

## Abstract

Brazil is among the world’s leading egg producers and currently has 1.4 million-layer breeders. Although genetic selection has improved reproductive traits more significantly in layer breeders than in their broiler congeners, aging is still an element that greatly affects fertility and hatch rate. Therefore, strategies to minimize age-related losses are crucial in the routine of layer breeder farms. Here, we assessed the effects of cloacal feather trimming (CFT) on reproductive parameters of a commercial-flock performing natural mating. To this end, a parent-stock house accommodating 11,238 hens and 1230 roosters was divided into 6 compartments, where 3 compartments containing roosters subjected to monthly CFT were interspersed with 3 compartments containing intact roosters. From 35 to 77 weeks of age, 2880 eggs from each group were collected every 40 days, marked and placed back with the rest of the eggs to undergo the company’s standard procedures. Afterwards, unhatched eggs from the two groups were subjected to embryodiagnosis. Aging had an impact on all the parameters evaluated, while CFT reduced infertility and early embryonic mortality, improving hatch rate and hatchability. Interactions between CFT and age were observed for infertility and early embryonic mortality. From 41 to 77 weeks of age, infertility in the CTF group decreased between 0.9 % and 3.3 % in relation to control group, whereas hatch rate increased between 2.6 % and 4.3 %. These findings indicate that a simple management such as CFT can significantly benefit the production of breeding flocks, and can be applied to the routine of parents, grandparents and great-grandparents.

## Introduction

Poultry farming has emerged as one of the most important production chains in Brazilian agribusiness, as a result of progressive investments in health, genetics, nutrition, welfare, and reproductive management (Vogado et al., 2016; Klein and Vidal, 2022). In this regard, commercial laying hens represent an important fraction of the national market since they provide the population an important source of high-quality protein at affordable prices (Amaral et al., 2016). Data from the Brazilian Animal Protein Association revealed that, in 2023, egg production was close to 52.5 billion units, 99 % of which was sold on the domestic market (ABPA, 2024). Besides, eggs have become an increasingly popular item on the Brazilian table with per capita consumption reaching 257 eggs in 2023 (ABPA, 2024). In general, genetic improvement in laying lines has resulted in breeders capable of sustaining high egg production and fertility rates for longer periods of time (averages ranging from 20 to 90 weeks of age) (Hy-Line, 2021; Lohmann, 2021; Hendrix-Genetics, 2022). In 2023, 1.39 million-layer breeders were housed generating a total of 130,659,228 commercial layers across the country (ABPA, 2024).

Given the importance of this niche in the poultry industry, research aimed at increasing the production of fertile eggs within a flock's reproductive cycle is becoming critical. In laying lines, peak production varies according to the breed, but generally occurs from the 25rd to the 55rd week with a tendency for a gradual reduction in both egg production and fertility as the flock ages (Hy-Line, 2021; Lohmann, 2021; Hendrix-Genetics, 2022). Therefore, the incorporation of approaches to mitigate these declines is interesting in order to guarantee flock profitability. Artificial insemination programs are routinely employed in the turkey industry and occasionally used in grandparent and great-grandparent chicken lines, and a common procedure in the preparation of roosters is cloacal feather trimming (CFT) (Dhama et al., 2014). This practice consists of cutting feathers around the cloaca in order to better visualize the area during semen collection, but it also reduces the loss of semen to the plumage or the contamination of samples by urine or feces that may be adhered to the feathers. This led us to hypothesize that the removal of feathers in the area would also facilitate the arrival of the semen into female reproductive tract during natural mating. However, to our knowledge, there are no systematic studies exploring the impact of CFT on the reproductive performance of layer or broiler breeders performing natural mating. Thus, we monitored a Bovans White parent flock from 35 to 77 weeks of age in which half of the roosters were subjected to monthly CTF and half were left intact, by comparing their reproductive indices under field conditions. Proof of the reproductive benefits of this management could lead to it being incorporated not only in layer or broiler parent stocks, but also at other levels of the poultry breeding chain such as grandparents and great-grandparents.

## Material and methods

### Experimental design

All procedures complied with the current regulations establish by the Ethics Committee of the College of Veterinary Medicine and Animal Sciences at the São Paulo

**Fig. 1:**
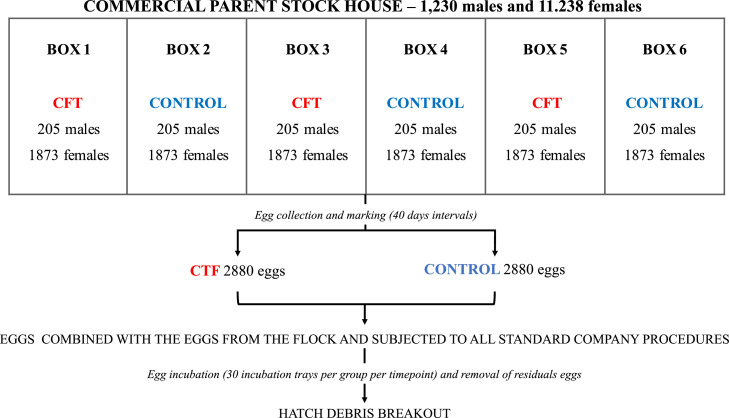
Diagram of the experimental design adopted in this study showing the distribution of the cloacal feather trimming (CFT) and control groups in the parent stock house (Bovans White from 33 to 77 weeks of age), and the egg collection, marking, management and analysis. At all experimental timepoints 30 incubation trays from each group containing 96 eggs were incubated, and subsequently hatch debris breakouts of all residual eggs were performed.

University (CEUAVET No 5477171122). This study was carried out in a parent stock house comprising 11,238 and 1,230 Bovans white hens and roosters, respectively, which were distributed in 6 compartments. Three compartments contained roosters subjected to monthly cloacal feather trimming (CTF group) and other three had roosters without any feather management (Control group) (Fig. 1). Cloacal feather trimming was performed every 4 weeks from 26 to 75 weeks of age in all individuals pertaining to the CTF group. The whole flock was exposed to the same rearing, feeding, ventilation, photoperiod and handling conditions throughout the study in order to avoid any environmental or management influences. Eggs were collected from 6 a.m. to 2 p.m. every 40 days, and marked with red and blue stamps for CTF and control groups, respectively (2880 eggs/ group/ collection). After marking, these eggs were returned to the boxes along with the other eggs from the flock.

All post-laying procedures followed the company’s standards both on the farm (i.e. egg collection, storage and transportation of the eggs) and in the hatchery (egg reception, selection, storage, pre-heating, incubation and hatching and processing of the chicks). At the hatchery the 2880 eggs from both groups were accommodated in incubation trays of 96 eggs (i.e. 30 trays from each group were incubated per timepoint). Then, following incubation, hatch debris breakouts were performed.

### Flock management

Throughout the production phase, males and females were kept on the floor on a bed of wood shavings which was only replaced during the sanitary break. Both sexes fed from the chain feeder systems (10 cm per bird) with feed being released four times a day (118 grams per bird). Chlorinated water (5 ppm) was provided *ad libitum* via nipple drinkers. Lighting was maintained at 16 hours of light per day (from 4:00 am to 8:00 pm) in an open-sided house equipped with yellow side-curtains, and the intensity of artificial light during dark periods was kept at 60 lux. The internal temperature in the house was set at 25°C, which was maintained by fans spaced 15 meters apart. The density of birds was 8 birds per square meter, and the density of manual nests was 4 birds per nest.

### Egg storage, processing and incubation

At the farm, the eggs were placed in plastic trays (30 eggs each) and stored in pallets (10,800 eggs) for 3 days at 20°C, and then transported at 19°C in specially designed egg trucks over a distance of 155 kilometers. As soon as they arrived, the egg pallets were placed in an egg storage room (18°*C* − 19°C), where they remained for up to 4 days. For preheating, eggs were removed from the plastic trays and accommodated in incubation trays (96 eggs) and trolleys, which were kept in front of the incubators inside the incubation room at a temperature of 26-28°C for 6-8 hours. Eggs were incubated using multistage setters with capacities for 124,416 eggs (model CASP CMG 124e, CASP, Brazil) with temperature and relative humidity between 99.3°F to 99.5°F and 80°F to 85°F (dry and wet bulb, respectively). Eggs were returned hourly until it was time to transfer them to the hatchers with capacity for 20,736 eggs (model CASP G21e, CASP, Brazil). Temperature and relative humidity in the hatchers ranged from 98.5°F to 97.5°F and 85°F to 89°F (dry and wet bulb, respectively). At 515 to 517 h of incubation, chicks were removed from the hatchers, selected, sexed, and the females were subcutaneously vaccinated (Marek and Gumboro) and placed in plastic transport boxes (100 chicks/ box) for shipment to customers. Unhatched eggs were opened and macroscopically categorized into the following groups: unfertile; embryonic mortality at 0-4 days; embryonic mortality at 5-10 days; embryonic mortality at 11-18 days; embryonic mortality at 18-21 days; pipped eggs; and contaminated eggs. Hatch rate (%) was calculated as the number of hatched chicks divided by incubated eggs and multiplied by 100, whereas hatchability (%) was calculated as the number of hatched chicks divided by fertile eggs and multiplied by 100. All hatch debris breakouts were performed by the same researcher throughout the study in order to avoid subjective variations in the results.

### Statistical analysis

All statistical analyses were performed using the Statistical Analysis System 9.4 software (SAS Institute, Cary, NC). Variables were initially tested to determine variance homogeneity and residual data normality by PROC GLM (Bartlett’s test) and PROC UNIVARIATE (Shapiro-Wilk test), and whenever these assumptions were not validated the data were transformed. The experimental unit were incubation trays of 96 eggs (30 trays per group per timepoint). The effects of treatment and flock age (week), as well as the interaction between both factors were estimated using the repeated-measures analysis of variance (Mixed Procedure of SAS). Then, comparisons between means of two groups were performed using t-test (PROC TTEST – SAS), whereas Fisher’s test (LSD) was applied for multiple comparisons. A confidence interval analysis was performed under the difference between control and treated groups, in which when confidence intervals of the difference between them did not overlap zero, the effect was considered statistically significant. All data are expressed as mean ± SEM with statistical significance set at *P* < 0.05.

## Results

**Fig. 2:**
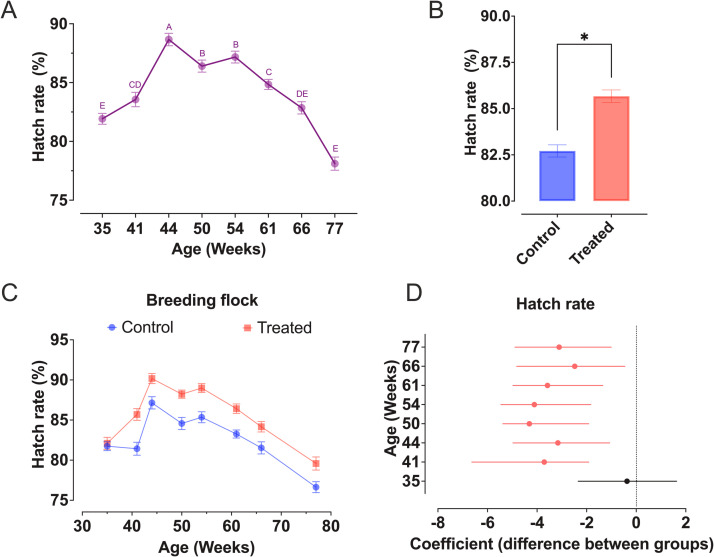
Effect of monthly cloacal feather trimming of the males (CTF) and flock age on hatch rate of layer breeders (Bovans White parent stock). (A) Changes in hatch rate from 33 to 77 weeks of age (different letters indicate significant differences among ages, *p* < 0.0001), (B) overall difference in hatch rate according to feather management (Treated = CTF group, asterisk indicate significant difference between groups, *p* < 0.0001), (C) changes in hatch rate in both control and Treated (CTF) groups throughout the monitoring, and (D) panel in which each point (i.e. difference in hatch rate between CTF and control groups) represent coefficient estimates from the fitted linear model, while solid lines represent 95 % confidence intervals. For any given coefficient estimate, it is considered significant (*p* < 0.05) whenever the confidence intervals do not cross zero (dashed lines).

**Fig. 3:**
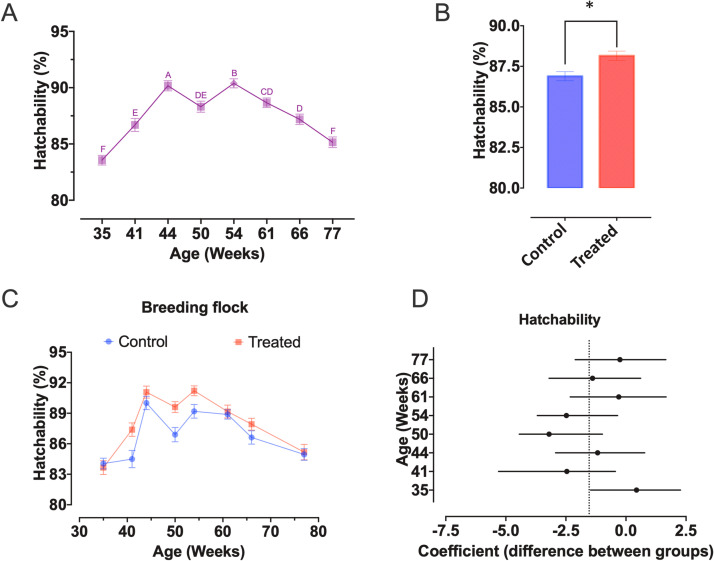
Effect of monthly cloacal feather trimming of the males (CTF) and flock age on hatchability of layer breeders (Bovans White parent stock). (A) Changes in hatchability from 33 to 77 weeks of age (different letters indicate significant differences among ages, *p* < 0.05), (B) overall difference in hatchability according to feather management (Treated = CTF group, asterisk indicate significant difference between groups, *p* = 0.0005), (C) changes in hatchability in both control and Treated (CTF) groups throughout the monitoring, and (D) panel in which each point (i.e. difference in hatchability between CTF and control groups) represent coefficient estimates from the fitted linear model, while solid lines represent 95 % confidence intervals. For any given coefficient estimate, it is considered significant (*p* < 0.05) whenever the confidence intervals do not cross zero (dashed lines).

**Fig. 4:**
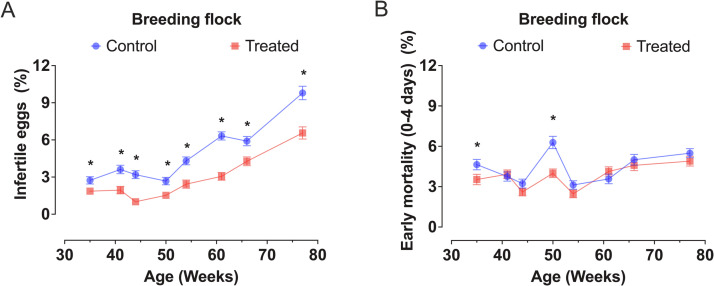
Means (± SEM) of (A) infertility and (B) early embryo mortality (0-4 days) of layer breeders (Bovans White parent stock) in which the roosters were submitted or not to monthly cloacal feather trimming (CTF/Treated and control groups, respectively). Asterisks indicate significant differences between control and treated (CFT) groups (*p* < 0.05).

Our analysis revealed that the age of the flock significantly affected all variables (Table 1), but it did not show a clear pattern of influence on the different parameters analyzed (i.e. with peaks and drops in each variable occurred at different ages). In contrast, we noticed that feather management had an impact only on infertility, early embryonic mortality (0-4 days), hatch rate and hatchability (Table 1). Besides, interactions between age and feather management were observed in relation to infertility and early embryonic mortality (0-4 days). Hatch rate peaked at 44 weeks of age (88.7 ± 0.5 %) and gradually decreased until 77 weeks of age (78.1 ± 0.6 %) (Fig. 2A), while the CTF as a whole improved this rate by 3.0 % (*p* < 0.0001 – Fig. 2B). All assessments, with the exception of the one at 35 weeks of age, confirmed significant differences in hatch rate between CTF and control groups (Fig. 2C and D). Hatchability also peaked at 44 weeks of age (90.1 ± 0.5 %) but remained above 88.3 % until 61 weeks of age, and the overall difference between CTF and control groups was 1.3 % (*p* = 0.0005 – Fig. 3A and B). However, the hatchability rates between CTF and control groups were not considered significantly different (Fig. 3C and D). Infertility in the control group was higher than CFT group throughout the study, with differences between groups varying from 0.9 % to 3.3 % (*p* < 0.05 – Fig. 4A). Higher early embryonic mortality (0-4 days) was observed in the control group only at 35 and 50 weeks of age (*p* < 0.05 – Fig. 4B).

**Table 1:** Probability of values for the effects of feather management (control and cloacal feather trimming) and flock age (Weeks) as well as the interaction of these factors for each variable assessed.

Variables (%)	Interaction effect		
	Feather Management	Age	Feather Management x Age
Contamination	0.2465	<.0001	0.0601
Infertility	**<.0001**	**<.0001**	**0.0023**
Mortality (0-4 days)	**0.0016**	**<.0001**	**0.0075**
Mortality (5-10 days)	0.2046	<.0001	0.9902
Mortality (11-18 days)	0.3057	<.0001	0.0836
Mortality (19-21 days)	0.5928	<.0001	0.2344
Pipped egg with live chick	0.7781	<.0001	0.1029
Pipped egg with dead chick	0.5027	<.0001	0.4303
Abnormal embryos	0.1230	<.0001	0.1129
Hatch rate	**<.0001**	**<.0001**	0.1849
Hatchability	**0.0005**	**<.0001**	0.0988

## Discussion

In the past decades, the layer breeding industry has undergone impressive changes with the aim of producing quality layers at suitable costs for the most diverse markets. However, despite layer breeds exhibit better reproductive performance than broiler breeds, it’s a common circumstance for both groups that the productivity of fertile eggs and chicks decreases with age (Tona et al., 2001; Iqbal et al., 2016; Bouba et al., 2021). Egg fertilization and embryo development in poultry depend on genetic and non-genetic factors linked to both roosters and hens, as well as the interaction between sexes (Wolc et al., 2009). Physiological issues that reduce semen quality or behavioral issues that affect the male’s ability to mate efficiently with females are the factors most associated with breeding roosters (Wolc et al., 2009). In this regard, higher weights or large amounts of breast fleshing can compromise the ability of males to mount and stay on females during insemination much more than in layer breeders, generating a more profound impact on fertility during aging. On the female side, the factors apparently critical to fertility and embryo survival are those connected to the prevalence of sperm storage tubules (SSTs) and internal and external egg quality traits (Wolc et al., 2009, 2010). Besides, it is very likely that during copulation part or all of the ejaculated semen is sometimes wasted into the pericloacal feathers, causing a decrease in the amount of sperm reaching the SSTs, and consequently losses in fertility. We therefore wanted to test whether trimming the feathers of this region in roosters would facilitate the deposition of semen in the cloaca of hens, generating improvements in the reproductive performance of a parent stock flock kept under commercial conditions. Considering the longer life cycle of layer breeders compared to broiler breeders and the added value of female chicks in this market, we believe that any management that increases fertility, and ultimately hatch rate, is interesting for the economic viability of genetic stocks.

As expected, at the end of our analyses we found that age impacted all the variables monitored, although these variables did not show similar variation trends over time. Infertility clearly increased as the flock aged, while hatch rate and hatchability initially rose but around 54 weeks of age began to fall. Nevertheless, the remaining parameters fluctuated without any pattern, such as a gradual increase during the monitoring. For instance, mortality rates at 5-10 days, 11-18 days and 19-21 days showed peaks at ages 41, 66 and 35 weeks, respectively. Previous studies, mainly with broiler breeders, have confirmed the influence of hen age on different hatching traits, although there was no consensus among researchers as to which hatching trait and period of time are most affected (Hocking and Bernard, 2000; Tona et al., 2001; Iqbal et al., 2016; Araújo et al., 2016). Here, most of the variables impacted by age are strongly dependent on egg quality and, in turn, on the hens such as contamination and embryonic mortalities. Yet we cannot rule out the role of farm, transport and hatchery managements in the fluctuations over time, even considering that the company’s standard procedures were always adopted during the study. This element could explain, for example, the unexpected rise in early embryonic mortality at 50 weeks of age regardless of the feather management (e.g. problems in the transportation or storage of eggs), or the increases in egg contamination seen at 35 and 77 weeks of age (e.g. poor nest cleaning or large numbers of floor eggs).

On the other hand, parameters such as infertility and early embryonic mortality and, consequently hatch rate and hatchability, exhibit a strong parental component or are related to the interaction between sexes. In this sense, a large body of data indicates that reproductive aging in roosters can affect both semen quality and sexual behavior through the following mechanisms: overweighting, testicular atrophy, epididymal lithiasis, compromised antioxidant system, reduced number of GnRH-I producing cells, reduced testicular LH and FSH receptors and reduced plasma FSH and testosterone concentrations (Ansari, 2024). Thus, any low-cost approaches that circumvent these barriers by increasing the number of complete matings or by increasing the number of sperm cells successfully delivered to the SSTs will be greatly accepted by poultry breeding farms. Our findings demonstrated CTF significantly reduced infertility throughout the study, achieving a 3.26 % and 3.23 % difference compared to the control group at 61 and 77 weeks of age, respectively. These results suggest that CTF effectively assisted the roosters in their task of depositing semen in the hen’s cloaca, which in turn had a strong impact on the hatch rate (improved overall by 2.96 %). Unfortunately, to our knowledge, there is no research in the literature that addressed this management so that we could compare results. With regard to early embryonic mortality, we found that the effect of CTF was not as pronounced as that observed in infertility with significant differences in relation to the control group occurring only at two ages (35 and 50 weeks of age), although the CTF had lower values at 6 of the 8 ages monitored. Previous studies have shown that supernumerary sperm have a functional role in early avian embryogenesis, since components present in avian spermatozoa seem to be responsible for triggering the progression of cell cycles during this phase (Wishart and Horrocks, 2000; Mizushima et al., 2014; Hemmings and Birkhead, 2015). Then, we believe that the greater delivery of spermatozoa to the SSTs also led to an increase in cells reaching the fertilization site in the infundibulum, ultimately causing an overall increase in hatchability (1.26 %).

Finally, another important aspect that needs to be discussed refers to economic and logistical aspects surrounding the implementation of CTF in commercial parent stock houses. This is because, depending on the country in question, the high labor costs may not be outweighed by the gains from additional chick production. When calculating the average egg production of the flock we monitored from 23 to 80 weeks of age (85 %), we estimate an average of 9,552 eggs per day (i.e., 286,561 eggs per month). The average hatch rate for this flock was 72 %, so considering that only female chicks are sold (half of the hatched eggs), we have 103,161 female chicks produced per month. Thus, if CTF increased the overall hatch rate by 3 %, we obtained a 1.5 % increase in the number of female chicks per month (1,547 birds) which, when sold for US$1 each (average prices at the time of the experiment in Brazil) generates a profit of US$1,547/ month. On the other hand, in terms of labor, 5 employees were needed for this management, working three extra hours for three days per month (one box per day). Considering that the cost of overtime at the company in question (including all charges) is US$12, we have a monthly cost of US$ 540, meaning that the profit from the sale of extra chicks exceeded the labor cost by almost three times. Hence, it is possible that in developing or undeveloped countries this type of management is profitable at the parent stock level. However, taking into account the values of the chicks produced by flocks of grandparents and great-grandparents, it is likely that this procedure will become economically attractive even for some developed countries.

In conclusion, we have shown that cloacal feather trimming (CTF) is a simple and cost-effective management that can be routinely used in poultry breeding farms to improve flock performance in terms of fertility, early embryonic mortality, hatch rate and hatchability.

## Data availability

The datasets generated during the current study are available from the corresponding authors on reasonable request.

## CRediT authorship contribution statement

**Eder O. Barbosa:** Writing – original draft, Project administration, Methodology, Data curation, Conceptualization. **Regina C.A. Porto:** Investigation, Data curation. **Marcel H. Blank:** Formal analysis. **Felipe L.K. Neto:** Investigation, Data curation. **Ricardo J.G. Pereira:** Writing – review & editing, Writing – original draft, Resources, Project administration, Methodology, Investigation, Funding acquisition, Formal analysis, Data curation, Conceptualization.

## Disclosures

The authors declare that they have no known competing financial interests or personal relationships that could have appeared to influence the work reported in this paper.

## References

[bib0001] ABPA (2024). Relatório Anual 2024 da Associação Brasileira de Proteína Animal. https://abpa-br.org/wp-content/uploads/2024/04/ABPA-Relatorio-Anual-2024_capa_frango.pdf.

[bib0002] Amaral G., Guimarães D., Nascimento J., Custodia S. (2016). Avicultura de postura: estrutura da cadeia produtiva, panorama do setor no Brasil e no mundo e o apoio do BNDES. BNDES Setor..

[bib0003] Ansari M. (2024). Recent strategies to mitigate reproductive aging in male broiler breeders: a review. Anim. Reprod. Sci..

[bib0004] Araújo I., Leandro N., Mesquita M., Café M., Mello H., Gonzales E. (2016). Effect of incubator type and broiler breeder age on hatchability and chick quality. Rev. Bras. Ciênc. Avíc..

[bib0005] Bouba I., Visser B., Kemp B., Rodenburg T.B., van den Brand H. (2021). Predicting hatchability of layer breeders and identifying effects of animal related and environmental factors. Poult. Sci..

[bib0006] Dhama K., Singh R.P., Karthik K., Chakrabort S., Tiwari R., Wani M.Y., Mohan J. (2014). Artificial insemination in poultry and possible transmission of infectious pathogens: a review. Asian J. Anim. Vet. Adv..

[bib0007] Hemmings N., Birkhead T.R. (2015). Polyspermy in birds: sperm numbers and embryo survival. Proc. R. Soc. B Biol. Sci..

[bib0008] Hendrix-Genetics (2022). Management guide parent stock. https://layinghens.hendrix-genetics.com/documents/1494/Management_Guide_Parent_Stock_EN_L2250-2.pdf.

[bib0009] Hocking P.M., Bernard R. (2000). Effects of the age of male and female broiler breeders on sexual behaviour, fertility and hatchability of eggs. Br. Poult. Sci..

[bib0010] Hy-Line (2021). Management guide - Hy-Line W-36 parent stock. https://www.hyline.com/filesimages/Hy-Line-Products/Hy-Line-Product-PDFs/W-36/36PSENG.pdf.

[bib0012] Iqbal J., Khan S.H., Mukhtar N., Ahmed T., Pasha R.A. (2016). Effects of egg size (weight) and age on hatching performance and chick quality of broiler breeder. J. Appl. Anim. Res..

[bib0013] Klein H., Vidal F. (2022). La emergencia de Brasil como principal exportador mundial de carne de pollo. Hist. Agrar. Am. Lat..

[bib0014] Lohmann (2021). Lohmann Brown and Lohmann LSL parent Stock Management Guide. https://lohmann-breeders.com/media/2020/07/LOHMANN-ParentStock.pdf.

[bib0015] Mizushima S., Hiyama G., Shiba K., Inaba K., Dohra H., Ono T., Shimada K., Sasanami T. (2014). The birth of quail chicks after intracytoplasmic sperm injection. Dev. Camb..

[bib0017] Tona K., Bamelis F., Coucke W., Bruggeman V., Decuypere E. (2001). Relationship between broiler Breeder’s age and egg weight loss and embryonic mortality during incubation in large-scale conditions. J. Appl. Poult. Res..

[bib0018] Vogado G.M.S., Vogado K.T.S., Fonseca W.J.L., Fonseca W.L., Oliveira A.M., Vogado W.F., Luz C.S.M. (2016). Evolução da Avicultura Brasileira. Nucl. Anim..

[bib0019] Wishart G.J., Horrocks A.J. (2000).

[bib0020] Wolc A., White I.M.S., Hill W.G., Olori V.E. (2010). Inheritance of hatchability in broiler chickens and its relationship to egg quality traits. Poult. Sci.

[bib0021] Wolc A., White I.M., Olori V.E., Hill W.G. (2009). Inheritance of fertility in broiler chickens. Genet. Sel. Evol..

